# Fabrication and biocompatibility assessment of polypyrrole/cobalt(II) metal-organic frameworks nanocomposites

**DOI:** 10.3906/kim-1910-63

**Published:** 2020-04-01

**Authors:** Mehrnaz MEHRABANI, Zeinab ANSARI-ASL, Farzaneh ROSTAMZADEH, Saeideh JAFARINEJAD-FARSANGI, Mahnaz Sadat HASHEMI, Mozhgan SHEIKHOLESLAMI, Zeinab NEISI

**Affiliations:** 1 Cardiovascular Research Centre, Institute of Basic and Clinical Physiology Sciences, Kerman University of Medical Sciences, Kerman Iran; 2 Department of Chemistry, Faculty of Science, Shahid Chamran University of Ahvaz, Ahvaz Iran; 3 Endocrinology and Metabolism Research Centre, Institute of Basic and Clinical Physiology Sciences, Kerman Iran; 4 Physiology Research Centre, Institute of Basic and Clinical Physiology Sciences, Kerman University of Medical Sciences, Kerman Iran; 5 Student Research Committee, Kerman University of Medical Sciences, Kerman Iran; 6 Herbal and Traditional Medicines Research Centre, Kerman University of Medical Sciences, Kerman Iran

**Keywords:** Nanocomposites, polypyrrole, metal-organic frameworks, cobalt

## Abstract

Nowadays, metal-organic frameworks (MOFs) have emerged as promising tools for different biological applications and therefore, efforts are ongoing to develop more biocompatible MOFs-based nanocomposites. We aimed to fabricate some new conductive nanocomposites of polypyrrole and cobalt-MOF with different weight percentages (PPy/x%Co-MOF) using the solution mixing method and characterize them through FT-IR (Fourier-transform infrared), PXRD (powder X-ray diffraction), SEM (scanning electron microscope), and TEM (transmission electron microscope) techniques. The biocompatibility of nanocomposites was assessed by haemolytic, cytotoxic, and quantitative reverse transcription PCR (qRT-PCR) assays. FT-IR and PXRD results revealed that nanocomposites consisted of pure MOFs and PPy. Moreover, SEM results indicated their spherical morphology along with an average diameter of 190 nm. (3-(4,5-Dimethylthiazol-2-yl)-2,5-diphenyltetrazolium bromide (MTT) assay showed a concentration, and percentagedependent cytotoxic effect of the nanocomposites on some cell lines including 3T3 fibroblasts, MCF-7, and J774.A1 macrophages. Haematological toxicity of PPy/x%Co-MOF composites was less than 7% in most concentrations. Furthermore, PPy/x%Co-MOF composites did not show any significant effect on the expression of cyclooxygenase−2( COX-2) and inducible nitric oxide synthase( iNOS) genes. In sum, regarding the haemolytic, proinflammatory, and cytotoxic tests, prepared nanocomposite demonstrated the reasonable in vitro biocompatibility which may be considered as a hopeful platform for further investigations including clinical applications.

## 1. Introduction

MOFs (metal-organic frameworks) are known as crystalline compounds produced from the metal ions and organic linkers. Because of their unique properties including high porosity, tunable chemical structure, and high surface area, MOFs have been used in various fields consisting of sensors, catalysis, biomedicine, and energy storage [1–5]. Wisser et al. fabricated Chitin-Cu-MOF composites and studied their application in airfiltration [6]. Li et al. reported the fabrication of γ -cyclodextrin and some MOFs composites as drug carriers [7]. However, there are some issues needed to be considered before practical application of MOFs including poor biocompatibility, short circulation time, and their low stability in the aqueous solution [8]. Furthermore, the presence of extensive void volume in the MOFs, similar to other porous structures results in a low mechanical strength [9]. The weakness of MOFs demands a combination of biocompatible polymers and MOF to improve fragility, solubility, stability, and porosity of the final product [8,10]. Some semiconductor polymers such as conductive polymers, polypyrrole (PPy), polythiophene (PTH), and polyaniline (PANI) contain unique features. These compounds, due to their excellent stability, electrical and mechanical properties, and lowcost preparations have been used in chemical sensors, membranes, photovoltaic cells, and biomedicine [11–14]. Chemical or electrochemical oxidation polymerization of monomers is considered as main routes for the synthesis of these conductive polymers [15,16]. Up to now, various composites of these polymers were fabricated and their potential applications were demonstrated. There are a few reports of MOFs with PPy. For instance, Jiao et al. introduced a composite manufactured by Zn/Ni-MOF and PPy for application in energy storage devices [17]. ZIF-PPy nanocomposites with outstanding capacitance for application in supercapacitor devices were synthesized by Xu et al [18].

Finally, the assessment of biocompatibility is a major necessity for as-prepared compounds, especially those considered for medical applications. Therefore, biological compatibility of different concentrations of PPy, Co-MOF, and PPy/x%Co-MOF composites were evaluated through in vitro cytotoxic, haemolysis, and inflammatory tests. Previous studies have used different cell types including both cancer and normal cell lines to evaluate the biocompatibility of MOFs. Chen et al. synthetized a MIL series MOF, MIL-100(Fe), and assessed its biocompatibility on 2 cancerous and normal liver cell lines and the results showed a desirable biocompatibility and low cytotoxicity [19]. Hopp et al. showed that zinc imidazolate framework 8 at concentration of ≤30 μg mL^-1^ had no significant cytotoxic effect on human metastatic breast cancer cells (MDA-MB-231), embryonic kidney cells (HEK 293), keratinocyte (HaCaT), mouse embryonic fibroblast cells (NIH/3T3), and macrophage cells (RAW 264.7) [20]. A previous study reported PPy@MIL-100 nanoparticle as a biocompatible pH- and near-IR-irradiation responsive carrier for doxorubicin hydrochloride [21]. It seems that cost-effectiveness and biocompatibility of nano-platforms make them suitable candidates for developing the novel therapeutic agents.

Therefore, the main objectives of this study were: (a) to synthesize and characterize Co and MOF nanocomposites, especially with conductive polymers and (b) to investigate their biological compatibility.

## 2. Results and discussion

### 2.1. Preparation and characterization of PPy/x%Co-MOF nanocomposites

**Figure 1:**
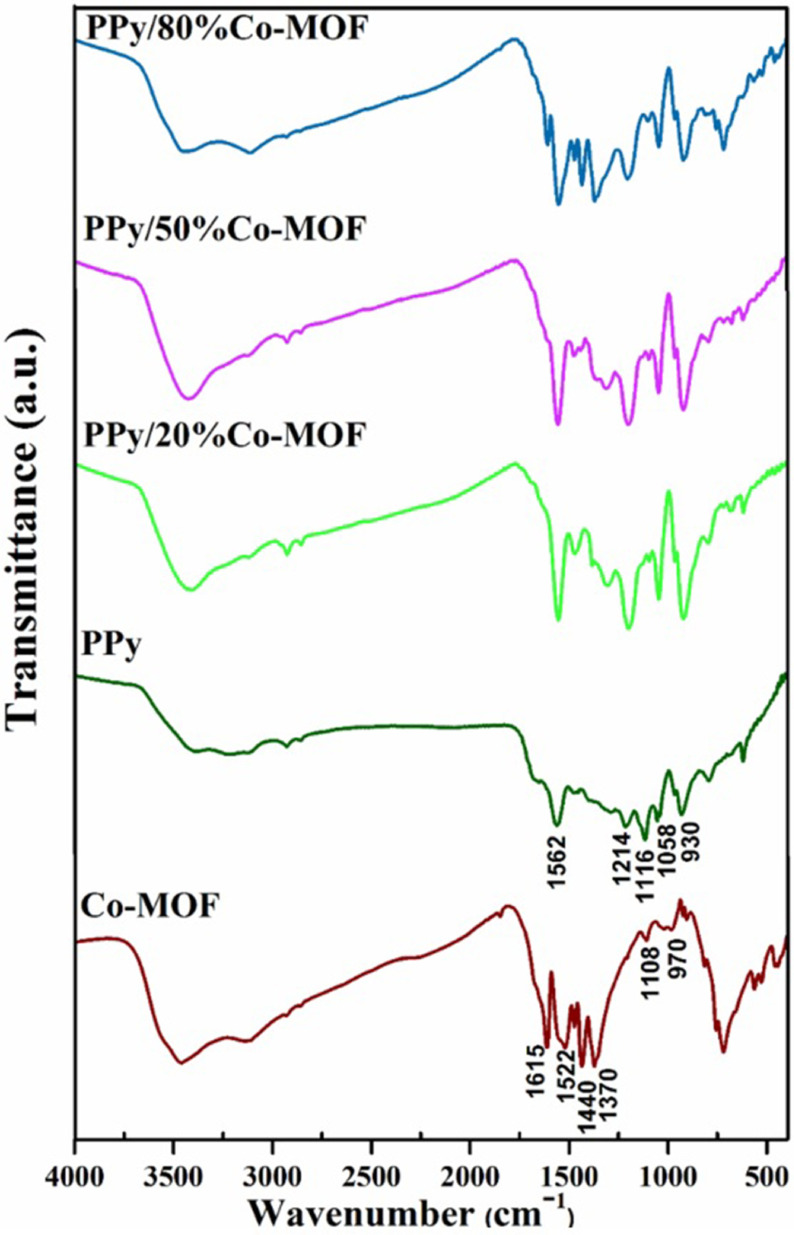
FT-IR spectra of Co-MOF, PPy, and PPy/x% Co-MOF nanocomposites.

In the last few years, MOFs have emerged as promising compounds owning to their specific characterization. Especially, transition-metal based metal-organic framework materials including Co-MOF have gained more attention because of their controllable structures, large surface areas, and adaptable pore sizes [22]. Up to now, different strategies have been proposed for modification of nanocomposites to overcome undesirable characteristics [23,24]. A 2-step process was used for the preparation of the PPy/x%Co-MOF composites, as given in the scheme. First, PPy and Co-MOF were synthesized using the chemical polymerization and solvothermal methods, respectively. In this way, the PPy/x%Co-MOF nanocomposites were fabricated by mixing Co-MOF and PPy solutions in NMP. The as-prepared compounds were then evaluated by some techniques such as FT-IR, PXRD, and SEM. The structure of nanocomposites was characterized by FT-IR spectra. Figure 1 shows the FT-IR spectra of Co-MOF and their nanocomposites with PPy. The PPy/x%Co-MOF composites indicated the characteristic peaks of the pure PPy and Co-MOF, which revealed the existence of these mentioned compounds in the as-formed nanocomposites. The characteristic absorption bands at 1562 cm^-1^ (C = C), 1116 cm^-1^ (C-C), and 930 cm^-1^ (C-H) were observed for pure PPy [25,26]. The Co-MOF FT-IR spectrum showed a distinctive peak at 1615 cm^-1^ which attributed to CO vibrational modes. The exhibited peaks at 1522 cm^-1^ , 1440 cm^-1^ , and 1370 cm^-1^ were ascribed to the symmetric and asymmetric stretching vibrations of the carboxyl groups. The observed peaks at 1108 cm^-1^ and 970 cm^-1^ were related to the C-N and N-CHO stretching vibrations, respectively, which confirmed the coordination of DMF to Co(II) [27–29]. The PPy/x%Co-MOF composites exhibited the characteristic peaks of the pure PPy and Co-MOF that confirmed the presence of these compounds in the as-formed nanocomposites.

**Scheme:**
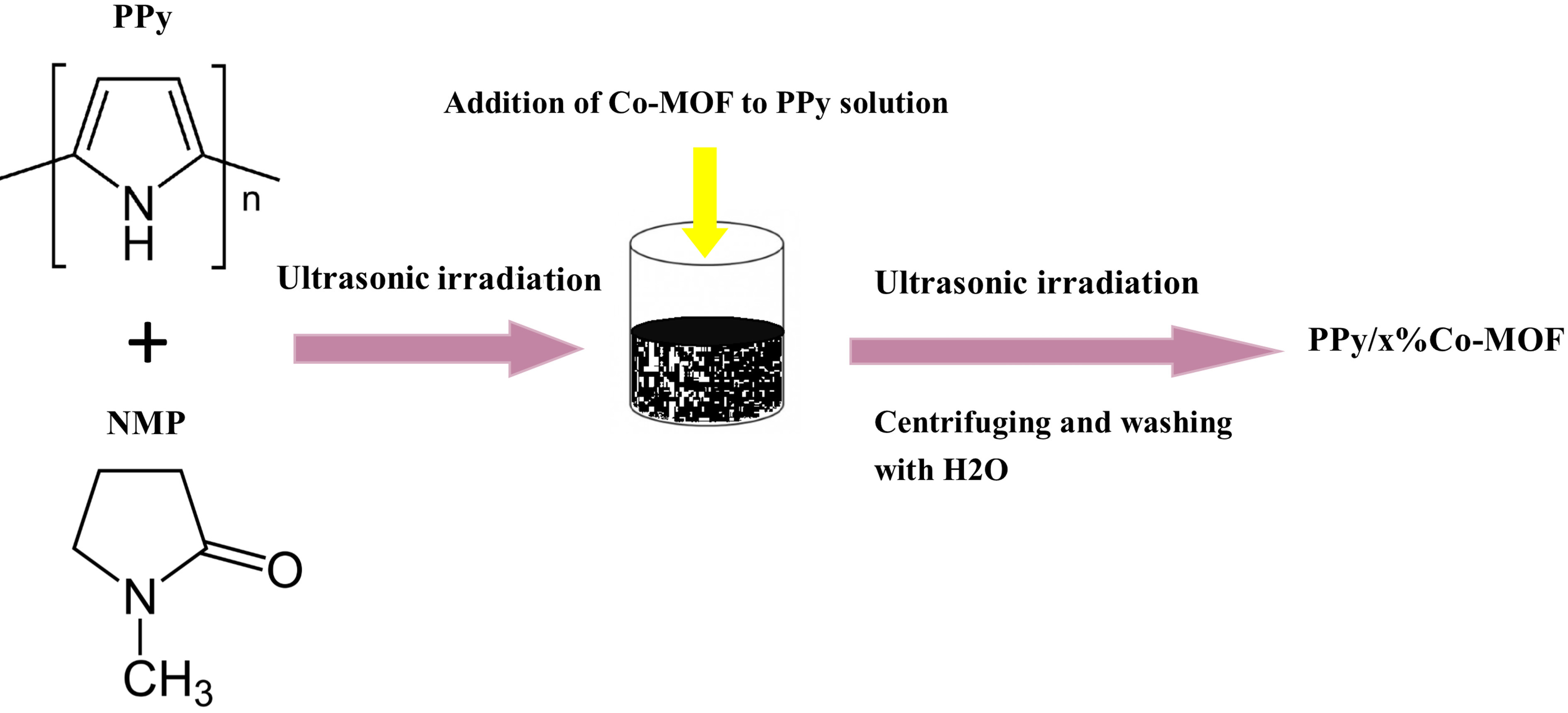
Schematic illustration of the fabrication process of PPy/x%Co-MOF nanocomposites.

**Figure 2:**
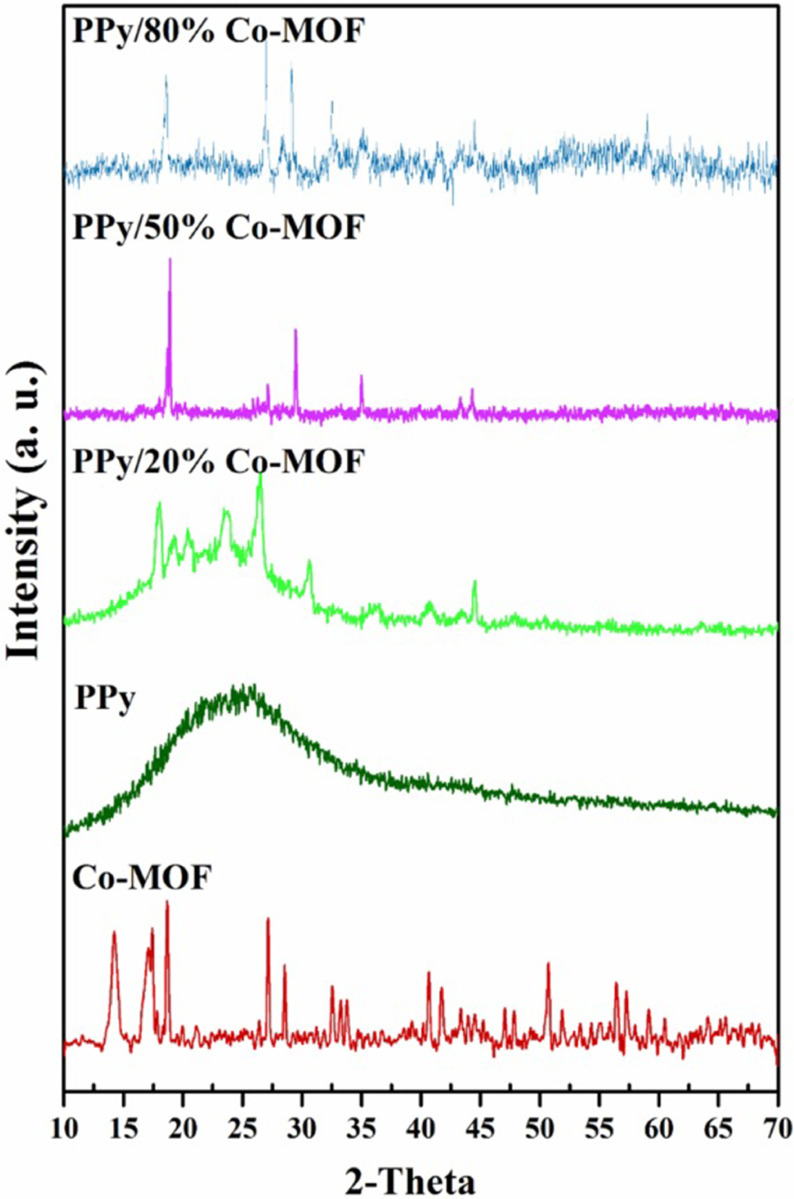
PXRD patterns of Co-MOF, PPy, and PPy/x% Co-MOF nanocomposites.

The microstructure of the as-prepared compounds was identified by PXRD analysis. Figure 2 shows the PXRD patterns of PPy, Co-MOF, and their nanocomposites. The PXRD pattern of PPy exhibited a broad peak at 2θ = 25, which confirmed its amorphous nature [30,31]. The appearance of sharp peaks in their PXRD patterns of Co-MOF confirmed its well-crystallized structure. The characteristic diffraction peaks of Co-MOF are listed in Table 1 [32,33].

**Table 1:** Characteristic XRD peaks and their corresponding crystallographic planes of Co-MOF.

2θ	14.2	17.4	18.6	27.1	28.5	29.2	32.5	33.3	33.7	34.8	36.6	41.7	43.3	43.9
(h k l)	(1 1 1^-^)	(2 2 0)	(1 1 1)	(2 0 2^-^)	(3 1 2^-^)	(0 0 2)	(0 2 2)	(3 3 1)	(6 2 1^-^)	(4 4 1^-^)	(4 4 0)	(4 4 1)	(1 1 3^-^)	(7 3 0)

**Figure 3:**
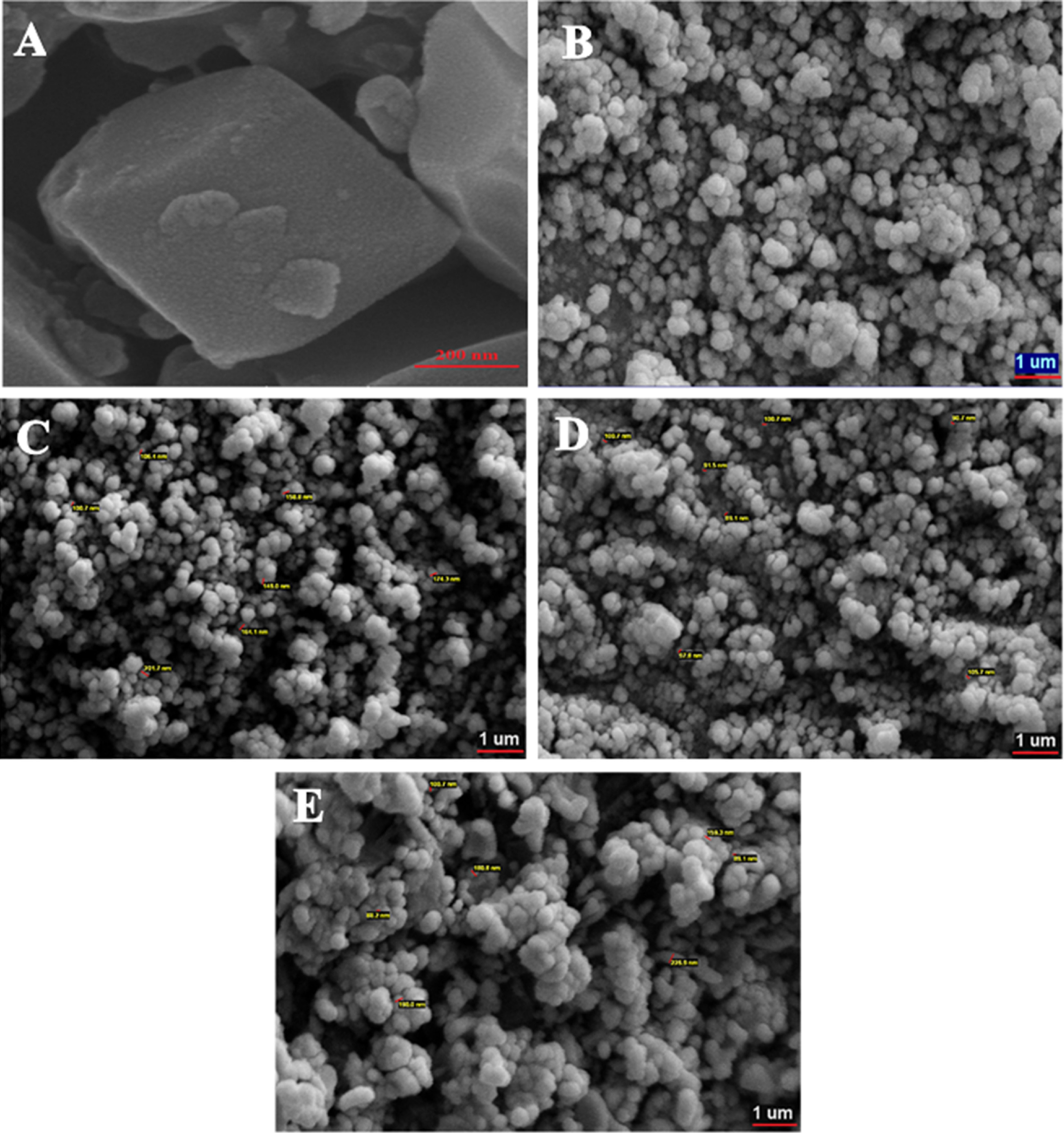
SEM images of (A) Co-MOF, (B) PPy, (C) PPy/20%Co-MOF, (D) PPy/50%Co-MOF, and (E) PPy/80%Co-MOF.

**Figure 4:**
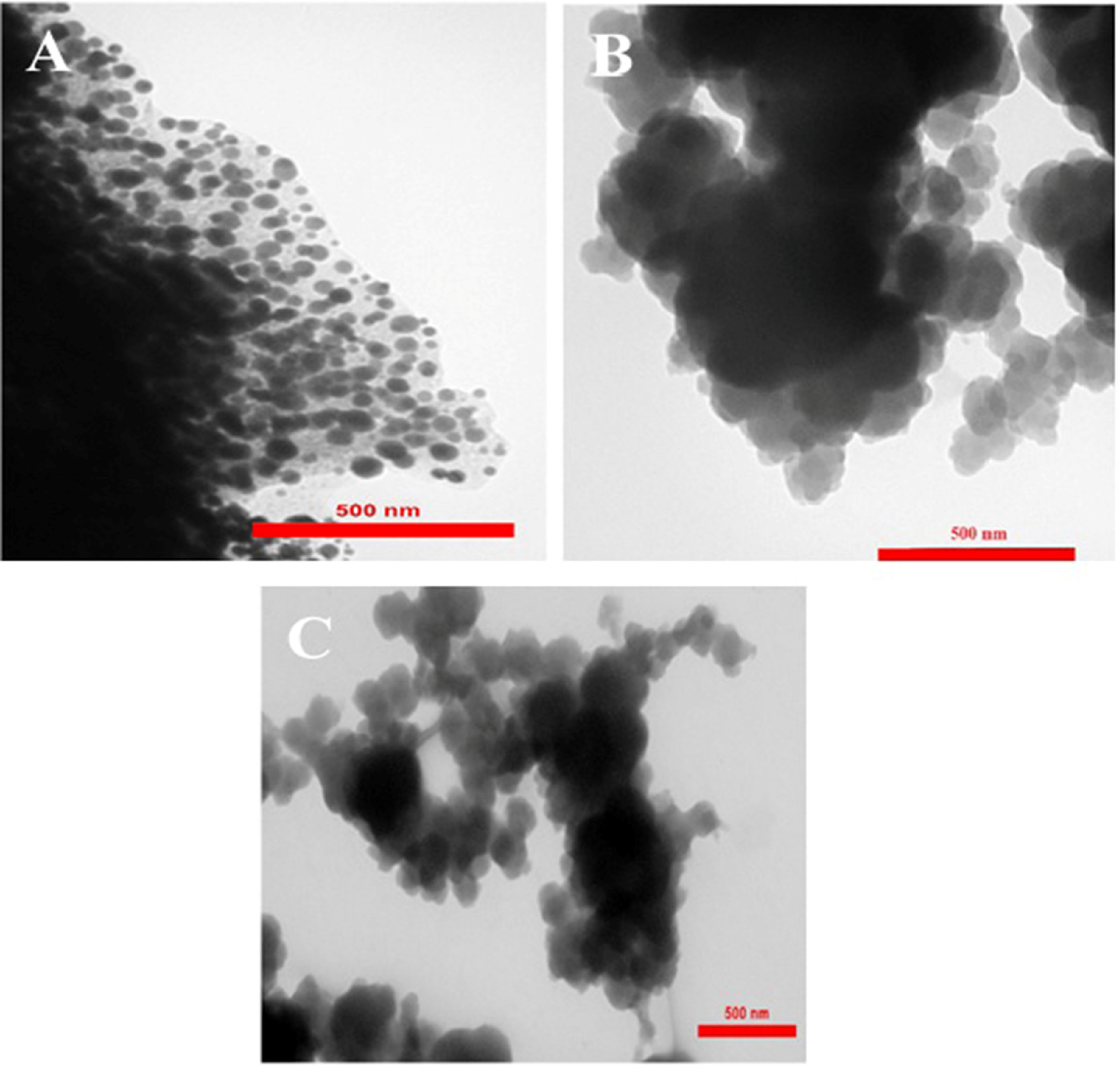
TEM images of (A) PPy/20%Co-MOF, (B) PPy/50%Co-MOF, and (C) PPy/80%Co-MOF.

SEM images of Co-MOF and PPy as well as their nanocomposites are depicted in Figure 3. It could be seen that PPy/x%Co-MOF nanocomposites exhibited a spherical structure with an average diameter of 190 nm. Therefore, the fabrication of nanocomposites did not have significant effects on the morphology of the pure PPy. Furthermore, it revealed that the diameter of nanocomposites is slightly higher than that of pure PPy. The morphological characteristics of the as-fabricated PPy/x%Co-MOF nanocomposites were also investigated by TEM. As depicted in Figure 4, TEM images of nanocomposites showed that the PPy/x%Co-MOF nanocomposites had a spherical structure with average diameters around 50 nm, 190 nm, and 220 nm for PPy/20%Co-MOF, PPy/50%Co-MOF, and PPy/80%Co-MOF, respectively. A small percentage of PPy in the PPy/20%Co-MOF and a lower aggregation between polymeric chains led to smaller nanospheres. In addition, based on the TEM images, it can be proposed that PPy can improve the stability of the Co-MOF by coating these particles.

### 2.2. Effect of PPy, Co-MOF, PPy/x%Co-MOF on the viability of 3T3 fibroblasts, J774.A1 macrophages and MCF-7 cells

There has been a trend toward using MOFs in biomedical fields. For example, MOFs is used as ideal vehicles for drug delivery. The ability to tune the porosity and adjust the structure makes MOFs suitable candidates for this target. MOFs are also used for nitric oxide storage as an important gas involved in numerous biological pathways. MOFs are also used for photodynamic therapies, sensing or bio-imaging agents [34]. Furthermore, it was shown that polypyrrole and its electrical composites were greatly researched for biomedical applications such as tissue regeneration, drug delivery systems, and electrical stimulation therapy owing to their low toxicity, ease of preparation, redox property, and high conductivity [35, 36].

In addition to all desirable characteristics of MOFs, there are some concerns about safety and biocompatibility of MOFs. Given that the interaction between such compounds and human cells is the most important aspect of biocompatibility tests. Therefore, we explored the effect of them on viability of three cell lines.

**Figure 5:**
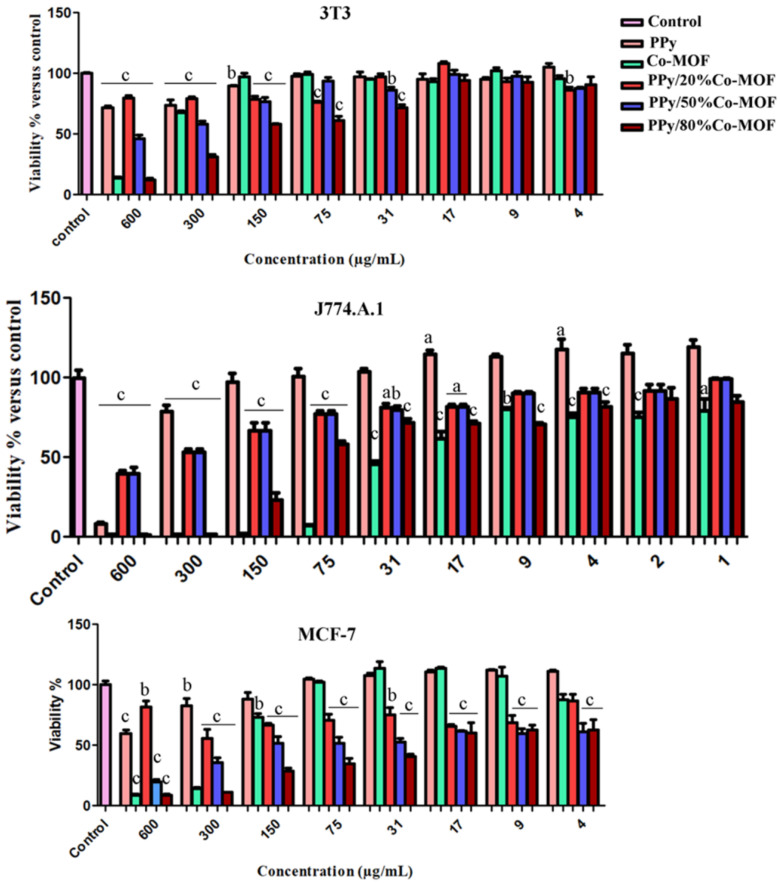
Evaluation of the effects of PPy/x%Co-MOF nanocomposites on the viability of 3T3, MCF-7, and J774.A1 cell lines by MTT assay. The viability percent of 3T3 (A), J774.A1 (B), and MCF-7 (C) cell lines were shown after treatment with different concentrations of Co-MOF, PPy, and PPy /x % Co-MOF. Results are reported as means ±SEM. (n = 6; *P <0.05 (a), **P <0.01 (b), ***P <0.001 (c) vs. control).

Previous reports showed that the toxicity of nanocomposites was highly related to their dosage, chemical composition, solubility, and surface chemistry [37]. As shown in Figure 5, PPy showed the highest nontoxic concentrations on 3T3; 75 (μg mL^-1^ ) , J774.A1; 150 (μg mL^-1^ ) and MCF-7; 150 (μg mL^-1^ ) between all treatments. Vaitkuviene et al. reported that PPy nanoparticles in a dose-dependent manner were cytotoxic at high concentrations, while they were biocompatible at low concentrations for Jurkat, MEF, and MH-22A cells [38]. The highest toxic effect was related to the effect of PPy/80%Co-MOF on 3T3 (31 μg mL^-1^) and PPy/80%Co-MOF on J774.A1 (4 μg mL^-1^) and PPy/50%Co-MOF and PPy/80%Co-MOF on MCF-7 (4 μg mL^-1^) . In a concentration-dependent manner, Co-MOF decreased the viability of all cell lines which was more significant in a J774.A1 cell line (lowest toxic concentration; 1 μg mL^-1^) . However, PPy/x%Co-MOF nanocomposites showed a lower toxic effect on macrophages than Co-MOF. A previous study has shown that the toxic effects of MOFs on macrophage cell line (RAW 264.7) were higher than those for the other cell lines [39]. Moreover, our results indicated that PPy/x%Co-MOF nanocomposites exerted higher toxic effects on cancerous cells compared to the other normal cell lines. The nontoxic concentrations of PPy/20%Co-MOF were 31 μg mL^-1^ in 3T3, 9 μg mL^-1^ in J774.A1 and 4 μg mL^-1^ in MCF-7. The nontoxic concentrations of PPy/50%Co-MOF were 17 μg mL^-1^ in 3T3 and 9 μg mL^-1^ in J774.A1. The nontoxic concentration of PPy/80%Co-MOF was 17 μg mL^-1^ in 3T3. PPy/50%Co-MOF and PPy/80%Co-MOF, even at the 4 μg mL^-1^ , showed a significant toxic effect on MCF-7. Some MOFs were considered as good candidates for in vivo applications, due to their low cytotoxicity accompanied with good biodegradability and biocompatibility [40].

### 2.3. The effect of nanocomposites on the mRNA levels of COX-2 and iNOS genes

**Figure 6:**
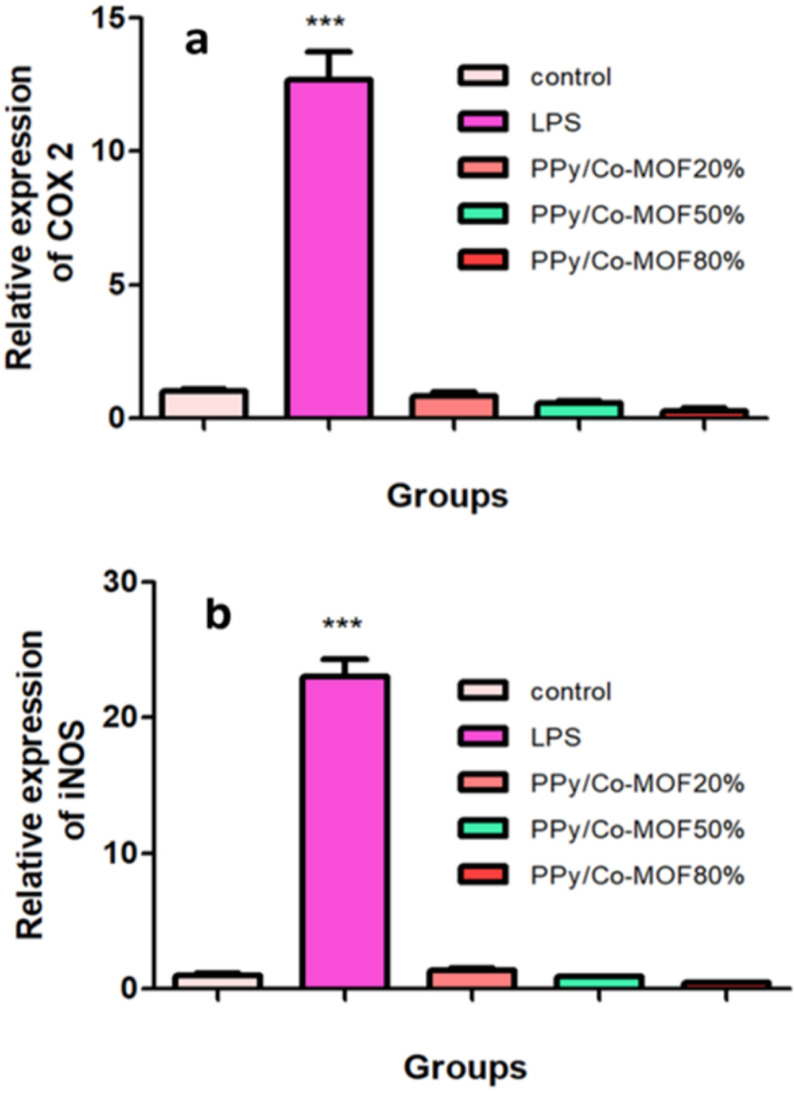
The effects of LPS and PPy/x%Co-MOF nanocomposites on the levels of COX-2 (a) and iNOS (b) genes expression. LPS significantly increased the expression level of both inflammatory genes. PPy/20%Co-MOF, PPy/50%Co- MOF, and PPy/80%Co-MOF did not influence iNOS and COX-2 expressions. All data are represented as mean ±SEM of 3 independent experiments (n = 3; *** P <0.001 vs. control group).

The non-toxic concentration did not change the COX-2 and iNOS mRNA levels in groups treated with nanocomposites (9 μg mL^-1^ for PPy/20% or 50%Co-MOF, and 2 μg mL^-1^ for PPy/80%Co-MOF) as compared with controls (Figure 6). The expression of proinflammatory genes was decreased in groups treated with PPy/x%Co- MOF nanocomposites, but it was not statistically significant compared to control. As reported in previous work, PPy/x%Cu-MOF nanocomposites did not change the expression of COX-2 and iNOS in J774.A1 cell line [41]. In response to inflammation challenge, expression of 2 important proinflammatory enzymes increased in macrophages as an important part of an innate immune system. COX-2 and iNOS are considered 2 inducible enzymes that are responsible for high levels of prostaglandins and NO production in inflamed site [42,43].

### 2.4. Effect of PPy/x%Co-MOF nanocomposites on the disruption of red blood cells

**Figure 7:**
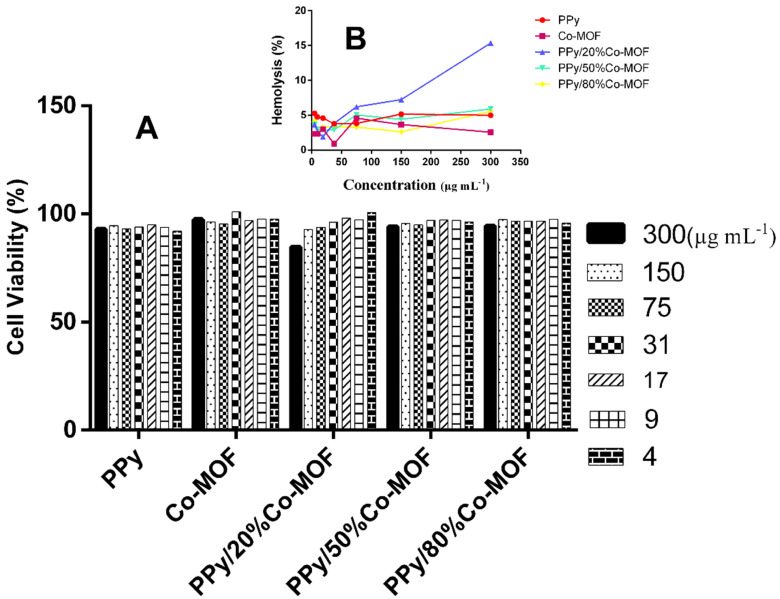
Cytotoxic effect of Co-MOF, PPy, PPy/x%Co-MOF nanocomposites on RBCs at different concentrations ranging from 4 to 300 μg mL^-1^ after 24 h. (A) percentage of viable RBCs and (B) percentage of haemolysis of RBCs.

Release of hemoglobin is a hallmark of material-derived toxicity of RBCs which can be detected by visible spectroscopy. We observed toxicity less than 7% for Co-MOF, PPy, PPy/x%Co-MOF on RBCs in all doses except for PPy/20%Co-MOF at 300 μg mL^-1^ which was 15% (Figure 7). Viability of RBCs after exposure to different concentrations up to 37 μg mL^-1^ of PPy/x%Co-MOFs was 97%.

## 3. Materials and methods

### 3.1. Materials

Cobalt (II) nitrate hexahydrate, dimethylformamide, ethanol, and ammonium persulfate (APS) were purchased from Merck Company. 1, 3, 5-benzenetricarboxylic acid, N-methyl-2-pyrrolidone (NMP), and Pyrrole were purchased from Sigma Aldrich Company. All cell culture materials were obtained from Gibco (Invitrogen, Carlsbad, CA, USA). 3-(4, 5-dimethylthiazol-2-yl)-2, 5-diphenyltetrazolium bromide (MTT) was obtained from Sigma (Sigma Aldrich, St Louis, MO, USA). RNX plus were purchased from CinnaGen (Tehran, Iran). Maxima SYBR Green/ROX qPCR master mix was from Fermentas (UK).

### 3.2. Preparation of Co-BTC metal-organic frameworks

Co-BTC was formulated using hydrothermal reaction of a mixture of 1,3,5-benzenetricarboxylic acid (0.005 mmol, 1.05 g) and cobalt (II) nitrate hexahydrate (0.01 mmol, 1.91 g) in 60 mL DMF:EtOH:H_2_ O (1:1:1) at 85 °C for 20 h in a stainless steel autoclave, based on the given procedure [44 ,45]. The products were separated by filtration, washed with EtOH, and dried at 60 °C for 10 h.

### 3.3. Preparation and characterization of PPy and PPy/x% Co-MOF nanocomposites

Polypyrrole was prepared based on our previous study [41]. Nanocomposites were obtained through the same synthetic procedure. Here, the detail of the PPy/80%Co-MOF is described. First, 0.4 g of Co-MOF was added to 10 mL of NMP including 0.1 g of polypyrrole. Then, the obtained mixture was ultrasonicated at room temperature for 2 h and stirred magnetically at 50 °C for 120 min. Afterward, the obtained product was centrifuged, washed with distilled H_2_O and EtOH, and dried in an oven at 70 °C for 24 h. Both PPy/50%Co- MOF and PPy/20%Co-MOF composites were fabricated by adding 0.1 g and 0.025 g of the Co-MOF to polypyrrole solution, respectively.

FTIR spectra of compounds were investigated as KBr pellets with an MB102 spectrophotometer. The produced crystalline structure was then examined through the Powder X-ray diffraction (PXRD) technique. SEM and TEM were employed to monitor the value of morphology of the generated composites.

### 3.4. Cell culture

Three cell lines including J774.A1 murine macrophage, MCF-7 human breast cancer, and 3T3 mouse embryo fibroblast were purchased from the Iranian Biological Research Center (Tehran, Iran). They were expanded in DMEM High Glucose (fibroblast and J774.A1 cells) and DMEM-F12 (MCF-7 cells). A humidified incubator was used for keeping them in 5% CO_2_ atmosphere at 37 °C conditions.

### 3.5. Cell viability assay

Among different methods used for evaluating cell viability, MTT is a simple and cost- effective colorimetric assay. In this assay, tetrazolium salt is reduced to purple formazan using dehydrogenase enzymes in the viable cells. 96-well culture plates were used for seeding the 3 cell lines at a density of 10,000 cells per well. Then, cells were treated with nanocomposites (4–600 μg mL^-1^) and kept in a humidified incubator for 24 h. In the last step, 10 μL of MTT dye (5 mg mL^-1^) was added to each well and plates were incubated at 37°C for 4 h. 100 μL of dimethyl sulfoxide (DMSO) was then added to each well. A microplate reader (Bio-Tek ELX800, USA) was applied to quantify the absorbance of samples at 570 nm [46].

### 3.6. Real-time PCR

J774.A1 cells were treated with media, bacterial lipopolysaccharide (LPS) (5 μg mL^-1^) as a positive control, and nanocomposites for 24 h. RNA was then extracted after incubation period by Rnx-plus (CinnaGen, Tehran, Iran) in accordance with the company’s recommendation. Complementary DNA was synthesized by PrimeScript 1st strand cDNA synthesis kit (Takara, Japan) in accordance with the manufacturer’s instructions. SYBR Premix Ex Taq II (Takara, Japan) was used to conduct real-time PCR for quantitative inducible nitric oxide synthase (iNOS), cyclooxygenase-2 (COX-2), and 18S ribosomal RNA(18S rRNA; internal control). Primer sequences were presented in Table 2. A StepOnePlus instrument (Applied Biosystems) was applied to perform all the reactions [47].

**Table 2:** Primers for real-time quantitative PCR.

Genes	Forward primer	Reverse primer
18 s	5’ GTAACCCGTTGAACCCCATT 3’	5’ CCATCCAATCGGTAGTAGCG 3’
iNOS	5’TGCATGGACCAGTATAAG GCAAGC 3’	5’GCTTCTGGTCGATGTCATAGCAA 3’
COX-2	5’ AACCGCATTGCCTCTGAAT 3’	5’ CATGTTCCAGGAGGATGGAG 3’

iNOS: inducible nitric oxide synthase. COX-2: cyclooxygenase-2.

### 3.7. Human red blood cells haemolysis

According to the previous study [41] for isolation of RBCs, 4 mL of whole blood was mixed with calcium and magnesium-free PBS in 2:1 ratio and centrifuged at 500 ×g for 10 min. After discarding the upper solution, RBCs pellets were centrifuged at 500 ×g for 10 min seven times with 10 mL PBS. Then, RBCs pellets were diluted by adding PBS up to 50 mL. Afterward, 800 μL of different concentrations (300, 150, 75, 31, 17, 9, 4 μg mL^-1^) of Cu-MOF, PPy, PPy/x%Co-MOF with 200 μL RBCs was used for spectroscopy. A positive control was distilled water and negative control was PBS. All samples were incubated in 37 °C for 3 h and were centrifuged to precipitate any debris at 10,016 ×g for 3 min [48]. The following formula was used for calculation of haemolysis percentage:

Haemolysis (%) =Dilluted RBCs abs540 − 655nm-PBS abs540−655nmDistilledwater abs540−655nm-PBS abs540−655nm

## 4. Conclusions

In summary, some new conductive composites of Co-MOF and polypyrrole were fabricated using a facile 2-step method. The porous Co-MOF was first prepared by hydrothermal procedure and then added to PPy solution in NMP. The as-formed nanocomposites exhibited spherical morphology. The structure and morphology of as-formed compounds were studied by various techniques including FT-IR, PXRD, SEM, and TEM. Then we explored a global effect of different concentrations of PPY/x%Co-MOF on 3 cell lines. In a concentrationdependent manner, PPy/x%Co-MOF reduced the viability of all cell lines and haemolysis. Furthermore, the highest toxic effect was related to PPy/80%Co-MOF. Totally, PPy/20%Co-MOF and PPy/50%Co-MOF were nontoxic at concentration lower than 9 μg mL^-1^ when considering both normal cell lines. Only PPy/20%Co- MOF at concentration of 4 μg mL^-1^ was nontoxic for MCF-7. Regarding PPy/80%Co-MOF, the concentration of ≤2 μg mL^-1^ was safe for normal cell lines. In conclusion, for further biomedical applications, the above nanocomposites might be considered as a promising platform at threshold concentration.
